# Cotton-Type Nanofiber Guided Pathway Engineering Enables Rapid Tissue Integration and Accelerated Bone Regeneration in Mineral Powder-Based Bone Grafts

**DOI:** 10.3390/jfb17040202

**Published:** 2026-04-20

**Authors:** Subin Park, Siphesihle Cassandra Nonjola, Jeong In Kim, Soonchul Lee

**Affiliations:** 1 Department of Orthopedic Surgery, CHA Bundang Medical Center, CHA University School of Medicine, 335 Pangyo-ro, Bundang-gu, Seongnam-si 13488, Republic of Korea; 2 Department of Anatomy, CHA University School of Medicine, 335 Pangyo-ro, Bundang-gu, Seongnam-si 13488, Republic of Korea; 3 SL Bio, Inc., 43, Beolmal-ro 30beon-gil, Bundang-gu, Seongnam-si 13503, Republic of Korea

**Keywords:** cotton-type nanofibers, electrospinning collector engineering, mineral powder–based bone grafts, tissue ingrowth pathways, bone healing

## Abstract

Mineral powder–based bone grafts exhibit excellent osteoconductivity; however, their clinical efficacy is often compromised by insufficient early-stage tissue ingrowth, leading to particle aggregation and pocket formation within the defect site during the initial healing phase. Here, we report a cotton-type nanofiber-guided mineral graft designed to overcome this early integration failure by creating fibrous pathways for tissue ingress. Cotton-type polycaprolactone (PCL) nanofibers were fabricated via electrospinning using a pin-based collector engineered to induce strong inter-fiber repulsion, resulting in a highly expanded, three-dimensional cottony architecture. Tetracalcium phosphate (TTCP) and α-tricalcium phosphate (α-TCP) mineral particles were subsequently deposited onto the surface of the cottony nanofibers, forming a fibrous–mineral hybrid graft (c-NF@T/α-TCP) in which the nanofibers act as a transient, functionally defined tissue-guiding framework during the early healing phase. The cottony nanofiber network effectively prevented mineral particle aggregation and generated continuous pathways within the graft, facilitating early tissue infiltration and vascular ingress during the first week after implantation. In vivo evaluation in a bone defect model demonstrated that c-NF@T/α-TCP significantly reduced tissue pocket formation at early time points and promoted subsequent bone regeneration compared to mineral powder-only grafts. This study highlights the critical importance of early-stage structural guidance in mineral-based bone grafts and introduces cotton-type nanofiber–guided pathway engineering as a simple yet effective strategy to unlock the regenerative potential of conventional inorganic bone substitutes.

## 1. Introduction

Mineral powder-based bone grafts, such as calcium phosphate ceramics, are widely used in clinical orthopedics and dentistry due to their excellent osteoconductivity and chemical similarity to native bone mineral [1,2,3,4]. However, despite their proven long-term biocompatibility, the regenerative outcomes of particulate inorganic grafts are often inconsistent, particularly during the early healing phase [5,6]. A critical limitation lies in the lack of structural guidance for early tissue infiltration, which frequently results in particle aggregation, soft-tissue encapsulation, and the formation of isolated pockets within the defect site [3,7,8]. These early-stage microenvironmental failures delay vascular invasion and subsequent osteogenesis, ultimately compromising the overall regenerative efficacy of mineral-based grafts [8,9,10].

Early tissue ingrowth is a decisive factor governing successful bone regeneration [11,12]. In the initial post-implantation period, mineral particles that are not rapidly integrated into host tissue tend to remain physically isolated, becoming trapped within fibrous soft tissue rather than participating in bone remodeling [3,5,12]. Although gradual dissolution and remodeling of calcium phosphate materials eventually occur, this process is often temporally mismatched with the biological demands of early regeneration, leading to prolonged healing times and incomplete defect repair [6,13,14,15]. Thus, strategies that actively promote early-stage tissue infiltration and vascular access are essential to unlock the full regenerative potential of inorganic bone scaffolds [1,8,12,16,17,18].

While composite grafts combining multiple calcium phosphate phases such as tetracalcium phosphate (TTCP) and α-tricalcium phosphate (α-TCP) are generally expected to outperform single-component systems by offering balanced resorption and mechanical stability, experimental observations often reveal the opposite trend. This discrepancy suggests that regenerative performance is often limited by structural constraints rather than material composition alone. In particular, α-TCP was selected in this study due to its relatively higher solubility and faster dissolution kinetics compared to other tricalcium phosphate polymorphs, such as β-TCP [19]. This rapid hydrolysis enables accelerated calcium ion release and apatite conversion, thereby enhancing early-stage tissue integration and vascularization compared to β-TCP. In addition, a TTCP/α-TCP biphasic system (1:1, *w*/*w*) was employed, combining rapid calcium ion release with hydrolysis-driven mineral transformation. Compared to more stable calcium phosphate phases such as hydroxyapatite or octacalcium phosphate, which exhibit slower dissolution kinetics, this biphasic composition is better suited to promote early-stage tissue integration. In practice, multiphase mineral systems can exacerbate early-stage tissue exclusion due to increased particle heterogeneity, packing density, and interparticle cohesion, further hindering tissue penetration [1,5,20,21,22,23]. Nevertheless, most existing studies continue to focus on compositional optimization of mineral phases, rather than addressing the fundamental structural barrier that limits early tissue ingrowth in particulate grafts [2,24,25,26,27,28,29].

Electrospun nanofibers have been extensively explored as tissue engineering scaffolds due to their extracellular matrix-mimicking morphology [23,30,31,32,33,34,35]. However, conventional electrospinning typically yields densely packed, two-dimensional or quasi-two-dimensional fibrous mats with limited thickness and pore interconnectivity [32,36,37,38,39,40,41,42]. Such structures, while suitable for surface-guided cell adhesion, are inherently insufficient for deep tissue infiltration when combined with mineral particulates. To overcome these limitations, we employed a modified electrospinning strategy to generate a highly expanded, three-dimensional cotton-type nanofibrous architecture. Unlike conventional electrospinning, which produces dense planar mats, this approach forms a continuous, volumetric fibrous network with high porosity and ECM-mimetic features. Compared to alternative fabrication methods such as freeze-drying, 3D printing, and foam replication—which lack nanoscale fibrous continuity or structural compliance—this system uniquely enables effective early tissue infiltration.

In this study, we introduce a cotton-type nanofiber guided mineral graft that fundamentally redefines the structural organization of fibrous mineral composites. By employing a custom-designed pin-based collector and precisely controlled temperature and humidity conditions, polycaprolactone (PCL) nanofibers were electrospun into a highly expanded, three-dimensional cottony architecture. This synergistic effect produces a volumetric, low-density nanofiber network resembling cotton candy, in stark contrast to conventional planar nanofiber mats.

When TTCP and α-TCP mineral particles are deposited onto this cotton-type nanofiber framework, the resulting fibrous-mineral hybrid graft forms continuous fibrous pathways throughout the mineral phase. These nanofiber-guided pathways prevent early particle aggregation, maintain open interstitial spaces, and actively facilitate tissue and vascular ingrowth during the critical first week after implantation. Rather than acting as a permanent load-bearing scaffold, the cotton-type nanofibers function as a transient, functionally defined tissue-guiding framework during the early healing phase, orchestrating early biological integration and becoming progressively replaced by regenerating bone.

By addressing the often-overlooked issue of early-stage structural guidance, this work demonstrates that pathway engineering via cotton-type nanofibers can dramatically improve the regenerative performance of conventional mineral powder grafts. Our findings highlight that, beyond material composition, the three-dimensional organization of graft components at the microscale is a key determinant of successful bone regeneration, and present a simple yet powerful strategy to overcome the intrinsic limitations of inorganic particulate bone substitutes.

## 2. Experimental Section

### 2.1. Materials Preparation

Poly(ε-caprolactone) (PCL; Mn = 80,000; CAS No. 24980-41-4) was purchased from Sigma-Aldrich (St. Louis, MO, USA) and used for the conversion process. Acetone (99.7%; CAS No. 67-64-1), dichloromethane (DCM, 99.8%; CAS No. 75-09-2) and lactic acid (LA; ≥90.0%; CAS No. 50-21-5) were obtained from Sigma-Aldrich (St. Louis, MO, USA). All chemicals were used as received without further purification. A 12 mL syringe fitted with a 20-gauge needle (20G) and polyethylene (PE) tubing (inner diameter, 1/16″; 1.6 mm) were purchased from Nano NC (Seoul, Republic of Korea). All cell culture reagents, including Dulbecco’s Modified Eagle’s Medium (DMEM), fetal bovine serum (FBS), trypsin, and penicillin/streptomycin were obtained from HyClone (Logan, UT, USA).

### 2.2. Preparation of TTCP, α-TCP, and T/α-TCP Composite Powders

TTCP and α-TCP powders were purchased from commercial suppliers and used as received. To prepare the composite inorganic phase, TTCP and α-TCP powders were mixed at a weight ratio of 1:1. The mixed powders were homogenized by ball milling to ensure uniform dispersion and intimate mixing of the two calcium phosphate phases. The ball-milled composite powder of TTCP/α-TCP composite (T/α-TCP) was subsequently collected and stored in a desiccator at room temperature prior to further use. The pristine TTCP, α-TCP, and the T/α-TCP composite powders were employed for scaffold mineralization as well as for in vitro and in vivo biological evaluations.

### 2.3. Fabrication of 3D c-NF and c-NF@T/α-TCP

A 10 wt% PCL solution was prepared by dissolving PCL pellets in a binary solvent system composed of DCM and acetone (2:1, *w*/*w*) under magnetic stirring until complete dissolution. LA was added to the as-prepared PCL solution at 10 wt% with respect to the PCL content to formulate PCL/LA composite solutions. Each solution was homogenized by continuous stirring prior to electrospinning. Three-dimensional (3D) cottony nanofiber (c-NF) structures were fabricated using a conventional electrospinning setup equipped with a pin-modified customized collector (p-mCC). The polymer solution was loaded into a plastic syringe connected to a stainless-steel spinneret via polymer tubing and delivered using a digital syringe pump. Electrospinning was performed at a flow rate of 1.0 mL h^−1^ under an applied voltage of 25 kV, with a fixed tip-to-collector distance of 15 cm. The spinning direction and all processing parameters were kept constant for all PCL/LA solutions to systematically assess the effect of LA incorporation on the formation of three-dimensional fibrous architectures. After electrospinning, the collected c-NF scaffolds were immersed in deionized (DI) water to remove residual lactic acid and solvent components. The electrospinning process was conducted in a temperature- and humidity-controlled environment maintained at 25 °C. A high relative humidity (>60%) was found to be critical for the formation of cotton-like three-dimensional nanofibrous structures, whereas under low humidity conditions (<35%), the fibers formed dense and planar architectures without volumetric expansion. The p-mCC consisted of stainless-steel pins mounted on a hemispherical stainless-steel mesh substrate, with all components obtained from local commercial suppliers. To enhance the focal concentration of the electric field during electrospinning, the peripheral edge of the collector was insulated using nonconductive adhesive tape. This collector configuration facilitated the formation of three-dimensional crumpled nanofibrous architectures. The washing process was continued until the pH of the collected wash solution reached neutrality (pH 7). The treated fibrous meshes were further rinsed with fresh DI water until the pH remained stable at neutral. The pH of the wash solution was monitored throughout the washing procedure using a calibrated pH meter. The mineral loading amount was controlled to 80 wt% with respect to the mass of the cottony nanofibrous scaffolds. The required amount of TTCP, α-TCP, or T/α-TCP composite powder was uniformly deposited onto the scaffolds, followed by drying at room temperature to obtain the cottony nanofibers decorated with tetracalcium phosphate/α-tricalcium phosphate mineral particles (c-NF@T/α-TCP). The mineral loading efficiency was quantified by gravimetric analysis based on the mass difference of the scaffolds before and after mineral deposition.

### 2.4. Morphological and Physicochemical Characterization of Scaffolds

The morphology of the inorganic minerals and nanofibrous scaffolds was examined using field-emission scanning electron microscopy (FE-SEM). The c-NF and c-NF@T/α-TCP were analyzed to evaluate the three-dimensional fibrous architecture, surface morphology, and deposition of inorganic mineral phases on the nanofiber surfaces. FE-SEM was further employed to verify the successful immobilization of TTCP and α-TCP particles on the fibers and to assess the particle size distribution and surface coverage of the mineral phases. The scaffold porosity and pore size distribution were quantified by image analysis of SEM micrographs using ImageJ software (version 1.54, National Institutes of Health, Bethesda, MD, USA) equipped with the ND image-processing plugin. SEM images acquired from multiple randomly selected regions of each sample were converted to binary images and processed using the ND plugin to extract pore size and pore spacing. In addition, the fiber diameter and the size of individual mineral particles were measured from FE-SEM images using ImageJ. The porosity-related parameters, fiber diameters, and mineral particle sizes were statistically evaluated based on measurements obtained from multiple independent micrographs for each sample.

X-ray diffraction (XRD) analysis was performed to confirm the crystalline phases of pristine TTCP, α-TCP, and the ball-milled TTCP/α-TCP composite, and to verify that the biphasic composite was formed without unintended phase transformation during processing. Diffraction patterns were collected using an XRD equipped with a Cu Kα radiation source (λ = 1.5406 Å) operated at standard voltage and current settings. Samples were scanned over a 2θ range of 10–60° with a step size of 0.02° at an appropriate scanning rate. The obtained diffractograms were compared with reference patterns to identify phase-specific reflections and to confirm the coexistence of TTCP and α-TCP peaks in the composite powder. Fourier-transform infrared spectroscopy (FT-IR) was performed to identify the chemical composition of pristine c-NF, TTCP, α-TCP, T/α-TCP, and c-NF@T/α-TCP, thereby confirming the successful incorporation of the inorganic components into the nanofibrous matrices.

To further investigate the three-dimensional architecture of the electrospun scaffolds, micro–computed tomography (micro-CT) analysis was performed. Scaffolds fabricated under low- and high-humidity conditions were scanned using a micro-CT system (SkyScan 1076, Bruker, Kontich, Belgium). Three-dimensional reconstructions were obtained to compare the structural differences between dense two-dimensional (2D) fibrous mats and highly expanded three-dimensional (3D) cotton-type architectures. The reconstructed images were analyzed to evaluate the volumetric distribution, pore interconnectivity, and overall spatial organization of the fibrous networks. These analyses enabled a direct comparison of the structural evolution of electrospun scaffolds as a function of humidity conditions.

### 2.5. PBS Immersion and SEM-EDS Analysis

To evaluate ion release behavior, c-NF@T/α-TCP scaffolds were immersed in phosphate-buffered saline (PBS; pH 7.4) at a concentration of 10 mg in 5 mL and incubated at 37 °C. At predetermined time points (1, 6, 12, and 24 h), samples were retrieved and dried overnight under ambient conditions. Elemental composition was analyzed using energy-dispersive X-ray spectroscopy (EDS) coupled with scanning electron microscopy (SEM; JSM-7800F, JEOL, Tokyo, Japan). For each sample, measurements were obtained from three randomly selected regions, and the average atomic percentages of calcium (Ca), oxygen (O), and phosphorus (P) were calculated.

### 2.6. Mechanical Testing

The compressive properties of the scaffolds were evaluated using specimens prepared with a uniform cylindrical geometry (diameter: 12 mm, height: 10 mm). Compression tests were conducted under dry conditions using a universal testing machine (Oriental Testing Machine, Oriental Testing M/C, version 12.7.2). Prior to testing, each sample was positioned to ensure proper alignment between the compression platens, and a preload was applied to establish full contact between the specimen and the loading surface. A constant crosshead speed of 2 mm/min was applied during the test, and the load–displacement data were recorded continuously. The compressive strength of the scaffolds was calculated based on the maximum load sustained by the specimen prior to failure, normalized to the cross-sectional area.

### 2.7. Cell Viability Assay

Human bone marrow–derived mesenchymal stem cells (hBMSCs; PCS-500-012, ATCC, Manassas, VA, USA) were expanded in Dulbecco’s Modified Eagle Medium (DMEM) supplemented with 20% fetal bovine serum (FBS) and 1% penicillin/streptomycin. Cells were maintained at 37 °C in a humidified atmosphere containing 5% CO_2_, and the culture medium was refreshed every two days. Extracts of α-TCP and/or TTCP were prepared in accordance with ISO 10993-12:2021 [43] at a concentration of 1 mg/mL in culture medium and incubated overnight to obtain the extraction media [44]. c-NFs were cut into uniform dimensions (1.2 cm × 1.2 cm) and sterilized by ultraviolet irradiation for 24 h. To prevent sample flotation during incubation, a sterilized rubber O-ring was placed over each matrix to ensure stable contact with the bottom surface of a 48-well culture plate (SPL Life Sciences, Pocheon-si, Republic of Korea). hBMSCs were seeded onto the matrices at a density of 2 × 10^4^ cells per well in 1 mL of complete DMEM. After 3 days of culture, cell metabolic activity and cytocompatibility were evaluated using a Cell Counting Kit-8 (CCK-8; Dojindo Laboratories, Kumamoto, Japan). Prior to analysis, the samples were gently rinsed with phosphate-buffered saline (PBS) to remove non-adherent cells. Subsequently, a mixture of culture medium and CCK-8 reagent at a 10:1 (*v*/*v*) ratio was added to each well and incubated for 2 h at 37 °C. The assay is based on the reduction of the water-soluble tetrazolium salt WST-8 to a colored formazan product by intracellular dehydrogenases in metabolically active cells. The absorbance was measured at 450 nm using a microplate reader (SpectraMax, Molecular Devices, San Jose, CA, USA), and the optical density values were used to quantify relative cell viability and proliferation.

### 2.8. Alkaline Phosphatase (ALP) Activity Assay

hBMSCs were seeded in 48-well plates at a density of 2 × 10^4^ cells/well and cultured for 10 days under osteogenic conditions. c-NF and/or inorganic material extracts (1 mg/mL) were applied during the culture period. Extracts of α-TCP and/or TTCP were prepared in accordance with ISO 10993-12:2021 using culture medium at an extraction ratio of 1 mg/mL, followed by overnight incubation [44]. After 10 days of culture, ALP activity was measured using a TRACP & ALP Assay Kit (MK301, Takara Bio Inc., Shiga, Japan) according to the manufacturer’s instructions with minor modifications. The culture medium was removed, and the cells were washed once with physiological saline (Daihan Pharm Co., Ltd., Ansan, Republic of Korea). Cells were then lysed by adding 150 μL/well of extraction solution (physiological saline containing 1% NP-40) and gently pipetted to ensure complete cell solubilization. An equal volume (150 μL/well) of substrate solution containing p-nitrophenyl phosphate (pNPP) prepared in ALP buffer (0.2 M Tris-HCl, pH 9.5, 1 mM MgCl_2_) was added, and the mixture was incubated at 37 °C in a CO_2_ incubator for 1 h. The enzymatic reaction was terminated by adding 150 μL/well of 0.5 N NaOH, which was prepared by dissolving sodium hydroxide pellets (Sigma-Aldrich, St. Louis, MO, USA) in distilled water. The absorbance was subsequently measured at 405 nm using a microplate reader, and ALP activity was quantified accordingly.

### 2.9. Cellular Morphological Analysis

Cellular morphological analysis was conducted to investigate the effects of mineral composition and fibrous architecture on hBMSC morphology. The experimental groups included control, TTCP, α-TCP, T/α-TCP, and c-NF@T/α-TCP. Extracts of α-TCP and/or TTCP were prepared in accordance with ISO 10993-12:2021 at a concentration of 1 mg/mL in culture medium and incubated overnight to obtain conditioned extraction media. For all groups, cells were cultured on coverslips. For the fibrous group, cotton-type nanofibers (with mineral coating) were wrapped onto the coverslips prior to cell seeding. hBMSCs were seeded at a density of 1 × 10^4^ cells per well for non-fibrous groups, whereas 2 × 10^4^ cells per well were seeded for the c-NF@T/α-TCP group. This adjustment was made to account for the three-dimensional architecture and increased effective surface area of the cotton-type scaffold, which requires a higher initial cell number to ensure comparable cell distribution and coverage across the scaffold volume. Importantly, this approach minimizes variability arising from uneven cell attachment and enables a more reliable comparison of cellular morphology across groups.

Cells were cultured in DMEM supplemented with the prepared extraction media under standard conditions (37 °C, 5% CO_2_, humidified atmosphere). After 3 days of culture, cells were fixed with 4% paraformaldehyde and permeabilized using 0.3% Triton X-100. The actin cytoskeleton was stained using F-actin–specific phalloidin conjugates (ReadyProbes™, Invitrogen, Waltham, MA, USA), and nuclei were counterstained with DAPI (Santa Cruz Biotechnology, Dallas, TX, USA). Fluorescence images were acquired using a confocal laser scanning microscope (LSM 880, Carl Zeiss, Oberkochen, Germany). Quantitative analysis of cell morphology was performed using ImageJ software (Ver. 1.54, NIH, Bethesda, MD, USA). Morphological parameters, including aspect ratio, circularity, and roundness, were calculated to assess hMSC spreading, elongation, and cytoskeletal organization.

### 2.10. Immunofluorescence Staining

Immunofluorescence staining was performed to evaluate endothelial activation and osteogenic differentiation under each experimental condition. Human umbilical vein endothelial cells (HUVECs) were used for CD31 staining, while hBMSC were used for RUNX2 staining. The experimental groups included control (CONT), TTCP, α-TCP, T/α-TCP, and c-NF@T/α-TCP. For the fibrous group, cotton-type nanofibers with mineral coating were prepared by wrapping onto the coverslips prior to cell seeding. Cells were seeded at a density of 1 × 10^4^ cells per well for non-fibrous groups, whereas 2 × 10^4^ cells per well were seeded for the c-NF@T/α-TCP group. This adjustment was introduced to account for the three-dimensional architecture and increased effective surface area of the cotton-type scaffold, ensuring uniform cell distribution and sufficient cell–material interactions throughout the scaffold volume, while minimizing variability caused by uneven cell attachment. The control group consisted of cells seeded on standard tissue culture-treated coverslips without any material treatment. HUVECs (CRL-1730, PCS-500-012, ATCC, Manassas, VA, USA) were cultured in Endothelial Growth Medium-2 BulletKit^®^ (EGM-2; CC-3162, Lonza, Basel, Switzerland). Cells were maintained at 37 °C in a humidified atmosphere containing 5% CO_2_. Extracts of α-TCP and/or TTCP were prepared in accordance with ISO 10993-12:2021 at a concentration of 1 mg/mL in culture medium and incubated overnight to obtain conditioned extraction media. Cells were cultured in their respective growth media supplemented with the extraction media where applicable.

After 3 days of culture, cells were fixed with 4% paraformaldehyde for 15 min and permeabilized using 0.3% Triton X-100 for 5 min. Non-specific binding was blocked by incubation with 1% bovine serum albumin (BSA) for 30 min. For endothelial analysis, HUVECs were incubated overnight at 4 °C with a primary antibody against CD31 (1:200; ab182981, Abcam, Cambridge, UK), followed by a 1 h incubation at room temperature with a goat anti-rabbit IgG H&L secondary antibody conjugated to Alexa Fluor^®^ 594 (1:1000; ab150080, Abcam, Cambridge, UK). For osteogenic analysis, BMMSCs were incubated with a primary antibody against RUNX2 (1:200; sc-390351, Santa Cruz Biotechnology, Dallas, TX, USA), followed by a donkey anti-mouse IgG (H+L) secondary antibody conjugated to Alexa Fluor™ 488 (1:1000; A21202, Invitrogen, Waltham, MA, USA). Nuclei were counterstained with DAPI. Fluorescence images were acquired using a confocal laser scanning microscope (LSM 880, Carl Zeiss, Oberkochen, Germany). Quantitative analysis of fluorescence intensity was performed using ImageJ software (Ver. 1.54, NIH, Bethesda, MD, USA), and mean fluorescence intensity (MFI) values were calculated to compare relative expression levels among different groups.

### 2.11. Rat Femoral Defect Model

Sixty male Sprague–Dawley rats (8 weeks old) were obtained from Koatech (Pyeongtaek, Republic of Korea) and acclimatized for one week prior to surgical intervention. The animals were 9 weeks of age at the time of surgery. General anesthesia was induced via intraperitoneal administration of a Zoletil and Rompun^®^ mixture (Zoletil: 0.4 mL/kg, Virbac Laboratories, Carros, France; Rompun^®^: 10 mg/kg, Bayer Korea Ltd., Seoul, Republic of Korea). After adequate anesthesia was confirmed, a longitudinal skin incision was made to expose the femoral shaft. A standardized cylindrical defect was created at the mid-diaphysis using a sterile 2 mm drill bit under aseptic conditions. Following implantation or defect preparation, the surgical site was closed using a Visistat^®^ Skin Stapler (Teleflex, Wayne, PA, USA). Postoperative monitoring was performed to ensure recovery and prevent complications. All animal experiments were conducted in accordance with institutional guidelines and were approved by the Institutional Animal Care and Use Committee (IACUC) of CHA University (Approval No. IACUC250138, Date: 25 August 2025).

### 2.12. Micro-Computed Tomography Evaluation

To assess bone regeneration and neovascular formation, animals were euthanized at 1 and 3 weeks postoperatively. The femora containing the defect sites were carefully harvested and subjected to micro-CT analysis using a SkyScan 1076 microfocus X-ray system (SkyScan^®^, Kontich, Belgium). Image acquisition and reconstruction were performed with NRecon Reconstruction^®^, and quantitative analyses were conducted using CTAn 1.8^®^ and CTVol software (version v.2.2. 1, Bruker, Kontich, Belgium). For 3D reconstruction, regions of interest (ROIs) corresponding to the defect area were precisely defined on two-dimensional cross-sectional images. Threshold values based on grayscale intensity were applied to distinguish mineralized tissue and vascular structures, enabling volumetric reconstruction of the selected regions. From the reconstructed 3D datasets, bone morphometric parameters—including bone volume fraction (BV/TV), trabecular thickness (Tb.Th), and trabecular number (Tb.N)—were quantified.

### 2.13. Histological and Immunohistochemical Analysis

Harvested femoral tissues were fixed in 4% paraformaldehyde at room temperature and decalcified in 17% (*w*/*v*) EDTA solution (pH 7.4) for 28 days with continuous mild agitation. The decalcified specimens were dehydrated through a graded ethanol series, cleared in xylene, embedded in paraffin, and sectioned into 7 μm-thick slices using a rotary microtome.

For general histomorphological evaluation, paraffin sections were deparaffinized in xylene and rehydrated through descending concentrations of ethanol (100%, 95%, 80%, and 70%) to distilled water. Sections were immersed in hematoxylin solution for nuclear staining, rinsed thoroughly under running tap water, and differentiated in acid alcohol if necessary. After bluing in alkaline solution, sections were counterstained with eosin to visualize cytoplasmic and extracellular components. The stained slides were subsequently dehydrated through graded ethanol, cleared in xylene, and mounted with a coverslip using a permanent mounting medium.

To assess collagen deposition and bone matrix formation, Masson’s trichrome staining was performed. Following deparaffinization and rehydration, sections were fixed in Bouin’s solution to enhance staining intensity and then rinsed in running water. Nuclei were stained with Weigert’s iron hematoxylin, followed by staining with Biebrich scarlet-acid fuchsin solution to label cytoplasmic elements. After differentiation in phosphomolybdic/phosphotungstic acid solution, sections were counterstained with aniline blue to visualize collagen fibers. Finally, sections were dehydrated, cleared, and mounted. Collagenous matrix appeared blue, while muscle and cytoplasm were stained red.

For immunohistochemical evaluation of osteogenic markers, deparaffinized and rehydrated sections underwent antigen retrieval in citrate buffer (pH 6.0) using heat-mediated treatment. Endogenous peroxidase activity was blocked with hydrogen peroxide, and nonspecific binding was minimized using a blocking solution prior to antibody incubation. Sections were incubated overnight at 4 °C with primary antibodies against RUNX2 (sc-390351, Santa Cruz Biotechnology) and osterix (ab22552, Abcam), CD31 (ab28364, Abcam), each diluted at 1:100. After washing, sections were treated with HRP-conjugated secondary antibodies and visualized using a DAB chromogen substrate kit (ab64261, Abcam) according to the manufacturer’s instructions. The sections were counterstained with hematoxylin, dehydrated, and mounted. All stained slides were scanned using a MoticEasyScan digital slide scanner (Motic Scientific, Universal City, TX, USA) for qualitative and quantitative assessment.

## 3. Results and Discussion

### 3.1. Mechanism of T/α-TCP-Decorated 3D Cottony Nanofibrous Scaffolds

**Figure 1:**
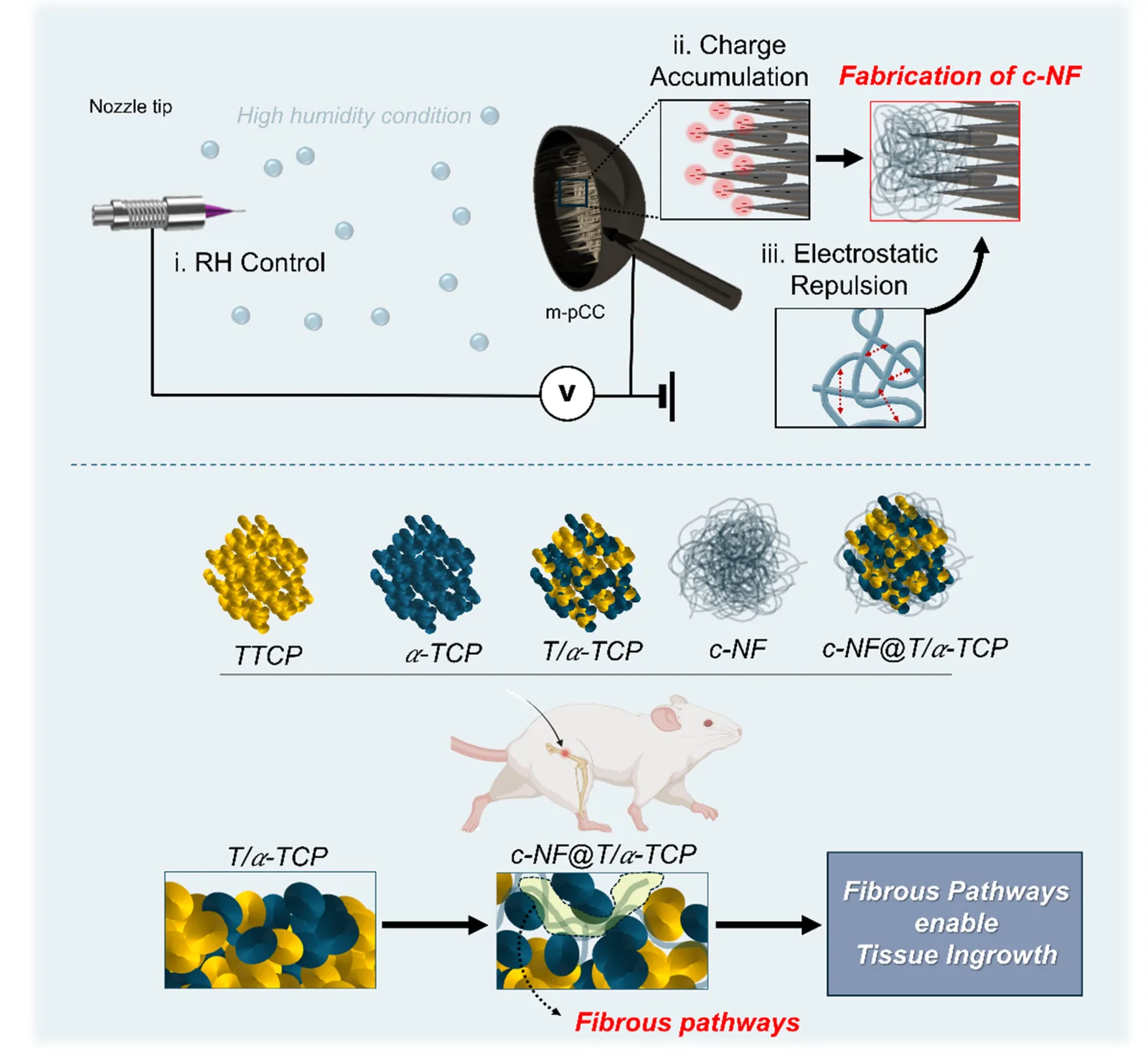
Fabrication and design concept of cotton-type nanofibers (c-NF) integrated with biphasic TTCP/α-TCP, forming a highly porous fibrous network that provides continuous pathways for tissue infiltration and mineral dispersion.

Figure 1 schematically illustrates the fabrication process of c-NF using a p-mCC and the subsequent design concept of integrating the fibrous scaffold with T/α-TCP composite minerals to promote tissue ingrowth through fibrous pathways. Unlike conventional planar collectors, the p-mCC generates a spatially heterogeneous electric field with multiple focal points around the protruded pins, which induces multidirectional stretching and deflection of electrospun jets as the charged polymer fibers approach the collector surface [45,46]. This non-uniform electric field distribution promotes random whipping and entanglement of fibers in three dimensions, preventing dense layer-by-layer stacking and instead facilitating the formation of a highly porous, crumpled, cotton-like architecture [46].

In addition to the collector-induced electrostatic focusing, the incorporation of LA into the PCL spinning solution plays a crucial role in driving the volumetric expansion of the nanofibrous assembly [46,47,48]. During electrospinning, LA molecules embedded within the PCL matrix partially dissociate, generating negatively charged carboxylate (–COO^−^) groups within the forming fibers. As solvent evaporation and polymer solidification proceed, electrostatic repulsion among these negatively charged moieties becomes increasingly pronounced [48,49]. This intrafiber and interfiber Coulombic repulsion induces spontaneous fiber separation and volumetric expansion, leading to a rapid “fluffing” behavior of the deposited fibers, reminiscent of cotton candy formation [49]. Consequently, the synergistic effect of (i) multidirectional electrostatic drawing by the p-mCC and (ii) charge-induced repulsive interactions originating from LA within the PCL fibers results in the formation of an ultralight, three-dimensional cottony nanofibrous architecture with interconnected macropores and open fibrous networks [45].

The resulting c-NF architecture was subsequently integrated with biphasic T/α-TCP composite minerals to construct a hybrid fibrous–inorganic scaffold. In contrast to mineral-only constructs, which mainly provide osteoconductive surfaces but lack interconnected fibrous guidance cues, the c-NF@T/α-TCP scaffold was designed to introduce continuous fibrous pathways that could potentially serve as physical conduits for cell migration and tissue infiltration. The highly porous cottony structure was intended to facilitate deep tissue penetration and neovascular invasion, while the immobilized calcium phosphate minerals were incorporated to provide localized osteogenic and bioactive cues. This hierarchical design strategy—combining mechanically compliant fibrous networks with osteoconductive inorganic phases—was conceptually proposed to address the limited tissue infiltration typically observed in densely packed mineral scaffolds, and to establish a microenvironment favorable for fibrous pathway-mediated tissue ingrowth and subsequent bone regeneration, which is systematically evaluated in the following sections.

### 3.2. Morphological and Structural Characterization of T/α-TCP-Decorated 3D Cottony Nanofibrous Scaffolds

**Figure 2:**
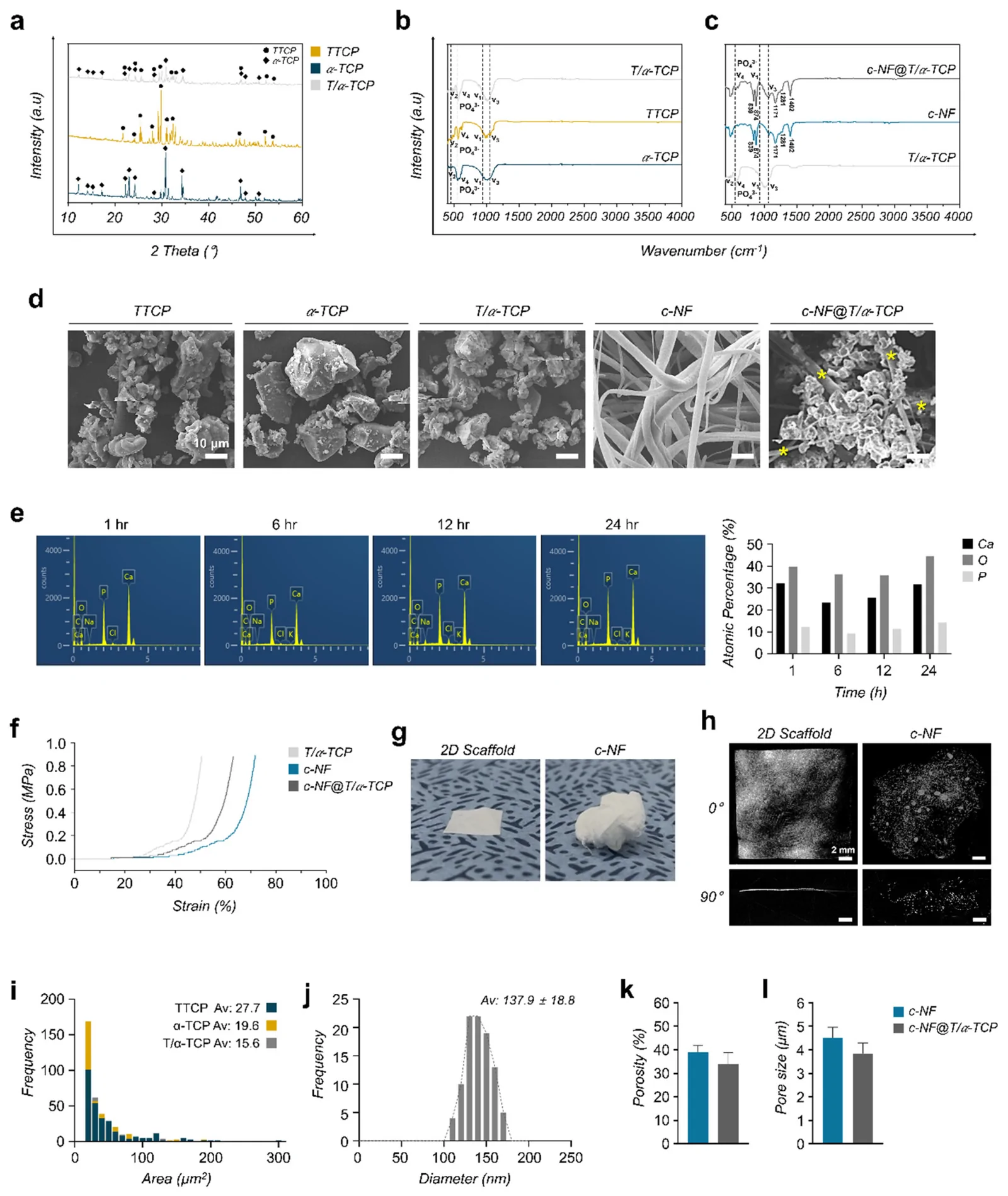
X-ray diffraction (XRD) analysis of TTCP, *α*-TCP and T/*α*-TCP showing the presence of both TTCP and *α*-TCP peaks in T/*α*-TCP sample (**a**). FTIR spectra of TTCP, α-TCP, and T/α-TCP (**b**), and c-NF and c-NF@T/α-TCP nanofibers (**c**). SEM images of TTCP, α-TCP, T/α-TCP, c-NF, and c-NF@T/α-TCP, where * indicates the presence of nanofibers (**d**). SEM-EDS analysis of c-NF@T/α-TCP after PBS immersion at 1, 6, 12, and 24 h (**e**). Stress–strain curves of T/α-TCP, c-NF, and c-NF@T/α-TCP scaffolds obtained from mechanical testing (**f**). Digital images of 2D scaffold and cotton-type nanofiber (c-NF) structure (**g**). Micro-CT images of 2D scaffold and c-NF structure at 0° and 90° orientations, showing orientation-dependent morphology (**h**). Particle size distribution of TTCP, *α*-TCP, and T/α-TCP (**i**). Diameter distribution of c-NF (**j**). Quantitative analysis of porosity (**k**) and pore size (**l**) of c-NF and c-NF@T/α-TCP scaffolds based on SEM image analysis.

Figure 2a shows the X-ray diffraction (XRD) patterns of TTCP, α-TCP, and ball-milled T/α-TCP to confirm their crystalline phase composition. The pristine TTCP and α-TCP powders exhibited their characteristic phase-specific diffraction peaks corresponding to their respective crystal structures. The XRD pattern of the T/α-TCP composite displayed the simultaneous presence of the representative reflections of both TTCP and α-TCP without the appearance of additional secondary phases. The preservation of these distinct phase-specific peaks indicates that the ball-milling process resulted in a physical biphasic mixture rather than inducing phase transformation or the formation of a new calcium phosphate phase, thereby confirming the successful fabrication of a TTCP/α-TCP biphasic system.

Figure 2b presents the FT-IR spectra of TTCP, α-TCP, and the T/α-TCP. Both TTCP and α-TCP exhibited characteristic phosphate (PO_4_^3−^) vibrational bands in the range of ~500–1200 cm^−1^, corresponding to ν_1_–ν_4_ stretching and bending modes of calcium phosphate phases [20,50,51,52]. TTCP and α-TCP showed slightly different peak positions and relative intensities of the PO_4_^3−^ bands, reflecting their distinct crystal structures and phosphate environments. The FT-IR spectrum of the T/α-TCP composite exhibited the simultaneous presence of characteristic PO_4_^3−^ bands originating from both TTCP and α-TCP, indicating that the two inorganic phases were successfully combined without phase transformation during the ball-milling process. The coexistence of these distinct phosphate-related vibrational features confirms the successful formation of a biphasic calcium phosphate composite, rather than the formation of a single new phase, which is desirable for achieving complementary dissolution kinetics and bioactivity.

Figure 2c compares the FT-IR spectra of c-NF, the T/α-TCP composite powder, and c-NF@T/α-TCP. The pristine c-NF exhibited the characteristic absorption bands of poly(ε-caprolactone) (PCL), including the C–O–C stretching vibrations at ~1170–1180 cm^−1^ and ~1240–1290 cm^−1^, the CH_2_ bending vibration at ~1400–1470 cm^−1^, and the crystalline phase-related band near ~840–875 cm^−1^ [53,54,55,56]. These peaks confirm the chemical integrity of the electrospun PCL nanofibrous scaffold. The T/α-TCP composite showed dominant phosphate-related bands characteristic of calcium phosphate minerals. Importantly, the FT-IR spectrum of c-NF@T/α-TCP exhibited both the characteristic PCL bands from the cottony nanofibers and the PO_4_^3−^ vibrational bands associated with the inorganic T/α-TCP phase. The coexistence of polymer- and mineral-derived peaks in the composite scaffold demonstrates the successful immobilization and deposition of T/α-TCP mineral particles onto the nanofibrous matrix without chemical degradation of the PCL backbone. These results confirm that (i) the biphasic T/α-TCP composite was successfully fabricated, and (ii) the inorganic mineral phase was effectively integrated onto the cottony nanofibrous scaffold, forming a hybrid architecture that combines the structural advantages of a three-dimensional fibrous network with the osteoconductive functionality of calcium phosphate minerals. This physicochemical integration is expected to provide favorable microenvironments for tissue ingrowth and bone regeneration by facilitating both cellular infiltration through the porous cottony structure and localized mineral-mediated osteogenic cues.

SEM micrographs of TTCP, α-TCP, T/α-TCP, c-NF, and c-NF@T/α-TCP are shown in Figure 2d. The pristine inorganic powders (TTCP and α-TCP) exhibited irregular particulate morphologies with broad size distributions, whereas the T/α-TCP composite displayed comparatively finer and more homogenized particle features as a result of the ball-milling process. This mechanical comminution and intimate mixing induced fragmentation of the original mineral agglomerates, yielding smaller and more uniformly distributed composite particles. Figure 2e presents the SEM–EDS analysis of the c-NF@T/α-TCP scaffold after incubation in phosphate-buffered saline (PBS) at different time points (1, 6, 12, and 24 h) to evaluate the stability of mineral immobilization under aqueous conditions. Elemental mapping and corresponding spectra consistently revealed the presence of calcium (Ca) and phosphorus (P) signals across all time points, indicating that the T/α-TCP mineral phase remained associated with the nanofibrous scaffold even after prolonged exposure to PBS. Quantitative analysis of the atomic composition showed slight variations in the relative Ca and P contents over time, which can be attributed to surface-level ionic exchange and partial dissolution–reprecipitation processes commonly observed in calcium phosphate materials under physiological conditions. Despite these dynamic changes, no significant loss of mineral components was detected, supporting the overall stability of the mineral coating. These results suggest that the mineral–fiber interface is stabilized by both electrostatic interactions and the three-dimensional nanofibrous architecture, which provides physical confinement and prevents detachment. Consequently, the c-NF@T/α-TCP scaffold exhibits a dynamic yet stable interface, enabling controlled mineral redistribution while maintaining sustained bioactivity under physiological conditions.

The compressive mechanical properties of the scaffolds were evaluated to investigate the effect of the fibrous architecture on structural stability. As shown in Figure 2f, distinct stress–strain behaviors were observed among the T/α-TCP, c-NF, and c-NF@T/α-TCP groups, reflecting their fundamentally different mechanical characteristics. The T/α-TCP scaffold exhibited a steep increase in stress at relatively low strain, followed by abrupt failure, indicating a typical brittle ceramic-like behavior [20,57]. In contrast, the c-NF scaffold demonstrated a gradual stress increase over a wide strain range, sustaining deformation up to ~70% strain, which is characteristic of a highly deformable polymeric network. In addition, the c-NF@T/α-TCP hybrid scaffold exhibited an intermediate mechanical response, combining the stiffness of the mineral phase with the deformability of the fibrous network. Compared to the T/α-TCP scaffold, the hybrid structure showed a delayed stress escalation and an extended strain range (~60%), indicating enhanced resistance to premature structural collapse. At the same time, it maintained higher stress levels than the c-NF scaffold at equivalent strain levels, suggesting effective load transfer between the mineral particles and the fibrous matrix.

This hybrid mechanical behavior reflects a transition from brittle to more ductile characteristics, which can be attributed to the presence of the cotton-type nanofiber network. The interconnected fibrous architecture likely redistributes applied stress and suppresses crack propagation within the mineral phase, thereby improving compressive resilience. These results demonstrate that the incorporation of a three-dimensional fibrous network significantly enhances the mechanical stability of mineral-based scaffolds. Such balanced mechanical properties are expected to be advantageous for maintaining structural integrity during the early stage of tissue infiltration and regeneration.

The macroscopic morphology of the scaffolds was further examined using digital imaging (Figure 2g). In contrast to the conventional 2D scaffold, which exhibited a compact and planar geometry, the c-NF displayed a highly expanded, cotton-like structure with a loosely packed and volumetrically enlarged morphology. This distinct configuration provides a direct visual representation of the three-dimensional fibrous assembly, complementing the SEM observations. To further evaluate the internal architecture and validate the three-dimensional porosity, Micro-CT analysis was performed (Figure 2h). The reconstructed images revealed a pronounced difference between scaffolds fabricated under different structural conditions. The conventional 2D scaffold exhibited a dense and compact structure with limited pore interconnectivity, whereas the c-NF showed a highly porous and interconnected network throughout the entire volume. The presence of continuous void spaces and well-developed fibrous pathways confirms the formation of an open and permeable three-dimensional architecture. Especially, orientation-dependent observations further emphasized this structural distinction, where the 2D scaffold consistently exhibited a planar morphology regardless of viewing direction, while the c-NF maintained its voluminous and porous three-dimensional structure even upon 90° rotation. These results demonstrate that the cotton-type nanofibrous scaffold possesses a uniquely expanded and highly interconnected porous structure. This structural feature is governed by relative humidity during fabrication, where scaffolds produced under high humidity conditions (>60%) form a highly expanded cotton-like architecture, whereas those fabricated under low humidity (30–40%) exhibit dense and planar structures with limited pore interconnectivity. Such humidity-dependent fiber expansion is expected to facilitate efficient cell infiltration and mass transport within the scaffold.

Quantitative particle size analysis was performed based on SEM images using ImageJ, and the corresponding size distribution profiles are presented in Figure 2i. The average particle sizes of TTCP, α-TCP, and T/α-TCP were determined to be 19.6, 27.7, and 15.6 μm, respectively. The reduced particle size of the composite powder relative to the pristine mineral phases reflects the effective size refinement achieved through ball milling, resulting in a more homogeneous composite inorganic phase. Although distinct differences in particle size distributions were observed among the three mineral formulations, all powders were subsequently applied at identical concentrations in the in vitro experiments. This experimental design minimized the influence of particle size as a dominant variable, allowing the biological responses to be primarily interpreted in terms of compositional effects rather than size-dependent artifacts. Figure 2j shows the diameter distribution of the electrospun c-NF scaffold. The cottony nanofibers exhibited a highly expanded three-dimensional morphology with minimal fiber–fiber contact points, indicative of a loosely packed fibrous network. The average fiber diameter was measured to be 137.9 μm, with a relatively narrow distribution, confirming the formation of a structurally uniform cotton-like architecture. Such a low-density, highly porous fibrous configuration is expected to provide extensive inter-fiber spaces and interconnected voids, which are advantageous for subsequent mineral deposition and potential tissue infiltration. In addition, in the c-NF@T/α-TCP construct, the mineral particles were observed to be uniformly immobilized on the external surfaces of the cottony nanofibers. The inorganic phases adhered to the fibrous matrix without apparent aggregation into large clusters, forming a discontinuous mineral coating along individual fibers. This interfacial assembly is attributed primarily to electrostatic interactions between the mineral particles and the surface of the nanofibers, facilitating stable attachment of T/α-TCP onto the fibrous framework. The resulting hybrid architecture preserves the open, three-dimensional fibrous morphology while simultaneously introducing osteoconductive mineral domains on the fiber surfaces, thereby establishing a hierarchical fibrous–inorganic microstructure. As observed in the SEM images (Figure 2d), the inorganic T/α-TCP particles were uniformly distributed on the fiber surfaces without disrupting the overall fibrous architecture. The mineral deposition process did not induce fiber bundling, pore collapse, or densification, thereby preserving the intrinsically open and highly expanded cotton-like morphology. Quantitative analysis of pore size and porosity based on SEM images using ImageJ revealed no statistically significant differences between c-NF and c-NF@T/α-TCP (Figure 2k,l). Both scaffolds exhibited comparably large pore sizes and high porosity, indicating that the introduction of inorganic mineral particles onto the fiber surfaces did not compromise the original macroporous characteristics of the cottony nanofibrous matrix (Figure 2k,l). This structural preservation is critical for the intended scaffold design, as it ensures the retention of interconnected void spaces that can serve as continuous pathways for tissue infiltration and mass transport [9,12,22,23]. These results demonstrate that mineral decoration of the cottony nanofibrous scaffold can be achieved without sacrificing its highly porous three-dimensional architecture. This feature supports the underlying design strategy of integrating osteoconductive inorganic phases into a mechanically compliant fibrous network while preserving the large, interconnected pore system required to facilitate deep tissue ingrowth and cellular infiltration after implantation.

### 3.3. Cell Viability and Early Osteogenic Response of Mineral–Fiber Hybrid Systems

**Figure 3:**
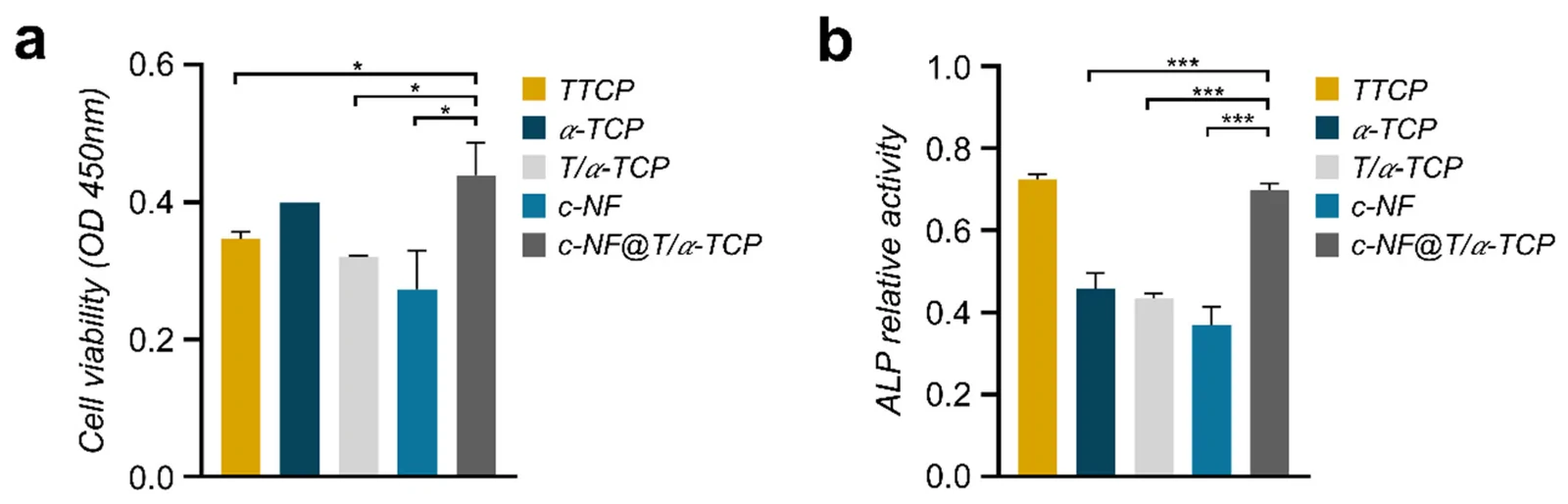
CCK assay showing cell viability of hBMSCs treated with mineral extracts or cultured on scaffolds (**a**). ALP activity indicating early osteogenic differentiation (**b**). Data are presented as mean ± SD. Statistical significance: * *p* < 0.05, *** *p* < 0.001.

The effects of calcium phosphate compositions and fibrous architecture on cellular responses were first evaluated by CCK assay (Figure 3a). Extracts of α-TCP and/or TTCP were prepared in accordance with ISO 10993-12:2021 using culture medium at an extraction ratio of 1 mg mL^−1^, followed by overnight incubation, and subsequently applied to the cells to assess the cytocompatibility of each mineral phase [44]. Among the mineral-only conditions, the biphasic system (T/α-TCP) exhibited slightly lower cell viability compared with the single-phase TTCP or α-TCP groups. In contrast, the c-NF showed the lowest cell viability, indicating that topographical stimulation by the polymeric fibers without bioactive mineral cues is insufficient to effectively support early cell activity. When the minerals were immobilized onto the fibrous network (c-NF@T/α-TCP), a significant increase in cell viability was observed, yielding the highest metabolic activity among all groups. This result suggests that the integration of bioactive mineral phases with the three-dimensional cotton-like architecture provides synergistic biochemical and biophysical stimulation. The expanded fibrous network likely improves cell attachment and spatial accommodation, while the surface-bound calcium phosphate phases provide localized osteoconductive ionic signals. Together, these combined cues enhance early cellular metabolic activity beyond what can be achieved by either mineral extracts or fibrous structures alone.

Early osteogenic differentiation was further assessed by ALP activity (Figure 3b). Among the mineral-only groups, TTCP induced significantly higher ALP activity than α-TCP or the biphasic T/α-TCP system, consistent with the well-known high calcium ion release and strong osteogenic stimulation associated with TTCP. Although TTCP demonstrated superior osteogenic potential as a single phase, its incorporation into the biphasic composition resulted in reduced ALP activity compared with TTCP alone. This decrease may reflect the moderated dissolution kinetics and attenuated ionic supersaturation in the mixed-phase system, which can dilute the strong osteogenic stimulus provided by TTCP. Consistent with the CCK results, the c-NF scaffold alone exhibited the lowest ALP activity, confirming that the fibrous topographical cue without mineral-derived biochemical signals provides minimal osteogenic induction. In contrast, the mineral-decorated fibrous scaffold (c-NF@T/α-TCP) showed a marked increase in ALP activity compared with the T/α-TCP mineral-only condition. Remarkably, the ALP level approached that of the TTCP single-phase group, despite the use of a biphasic mineral composition.

This enhanced osteogenic response highlights the critical role of structural context in modulating the biological performance of calcium phosphate materials. The cotton-type nanofiber architecture likely facilitates efficient cell infiltration, stable cell–material interactions, and localized mineral presentation, thereby amplifying the biological efficacy of the deposited phases. Rather than relying solely on compositional optimization, these results demonstrate that spatial organization of minerals within a highly expanded fibrous framework can restore or even potentiate osteogenic signaling. Overall, the combined findings from Figure 3a,b indicate that while multiphase calcium phosphate systems alone may exhibit moderated biological activity, their integration within a three-dimensional cotton-type nanofiber matrix generates a synergistic effect, simultaneously enhancing cell viability and early osteogenic differentiation. This mineral–fiber hybrid strategy effectively couples biochemical osteoconductivity with structural guidance, providing a promising design principle for next-generation bone graft materials.

### 3.4. Immunofluorescence Evaluation of Cellular Responses in Mineral–Fiber Hybrid Scaffolds

**Figure 4:**
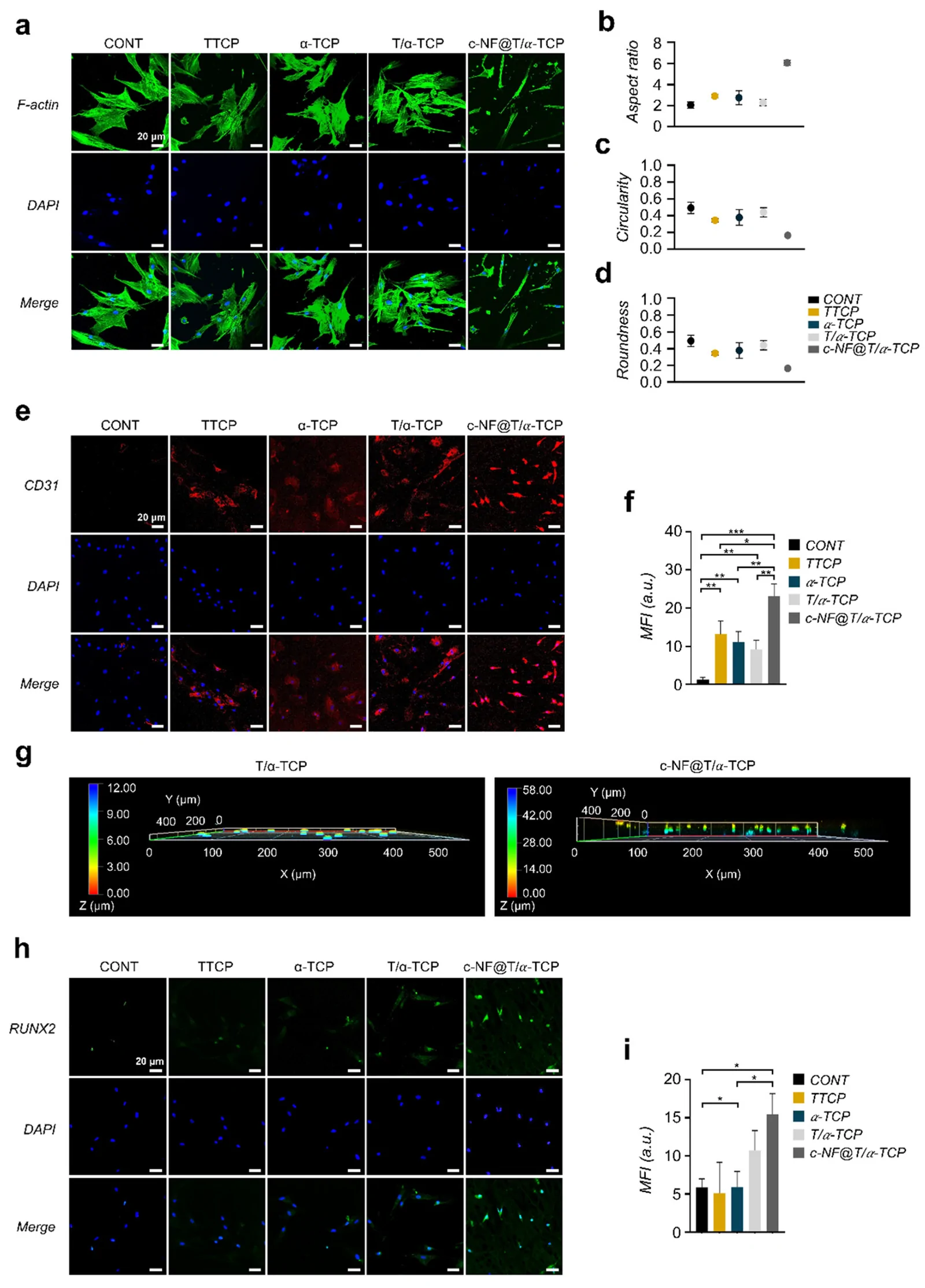
Fluorescence images of F-actin (green) and DAPI (blue) staining of hBMSCs cultured on different groups (CONT: cells cultured on tissue culture-treated coverslips without mineral-based grafts or c-NF) (**a**), with quantitative analysis of aspect ratio (**b**), circularity (**c**), and roundness (**d**). Immunofluorescence staining of CD31 (red) and DAPI (blue) in HUVECs (**e**), with quantitative analysis of mean fluorescence intensity (MFI) (**f**). Z-axis reconstruction and false-color depth mapping of DAPI-stained cells (**g**). Immunofluorescence staining of RUNX2 (green) and DAPI (blue) in hBMSCs (**h**), with quantitative analysis of mean fluorescence intensity (MFI) (**i**). Data are presented as mean ± SD. Statistical significance: * *p* < 0.05, ** *p* < 0.01, *** *p* < 0.001.

Figure 4 presents immunofluorescence analysis of cell morphology, endothelial activation, and osteogenic marker expression to further elucidate the mechanisms underlying the enhanced biological responses observed in the mineral–fiber hybrid system. Cell morphology and cytoskeletal organization were first assessed by F-actin and DAPI staining (Figure 4a). Cells cultured on c-NF@T/α-TCP exhibited a markedly elongated morphology with pronounced cytoskeletal alignment along the fibrous structures, whereas cells in the control and mineral-only groups displayed a more spread and isotropic morphology. Quantitative analysis revealed a significant increase in aspect ratio and a decrease in circularity and roundness in the fiber-containing groups (Figure 4b–d), indicating that the cotton-type nanofibrous architecture provides strong topographical guidance cues that promote cell elongation and alignment. In contrast, mineral-only groups did not show significant changes in morphological parameters, suggesting that biochemical cues alone are insufficient to induce pronounced cytoskeletal reorganization. Endothelial activation was further evaluated by CD31 immunostaining (Figure 4e). Mineral-treated groups (TTCP, α-TCP, and T/α-TCP) exhibited increased CD31 expression compared with the control, indicating that calcium phosphate-derived ionic signaling contributes to angiogenic activation. The c-NF@T/α-TCP group showed the highest CD31 expression among all groups. Quantitative analysis of mean fluorescence intensity (MFI) confirmed this trend (Figure 4f), suggesting that the combination of fibrous architecture and mineral-derived biochemical cues synergistically enhances endothelial responses.

To further investigate the physical effect of the fibrous architecture on cellular behavior, z-axis reconstruction combined with DAPI staining and false-color depth mapping was performed (Figure 4g). Cells cultured on the c-NF@T/α-TCP group exhibited markedly enhanced penetration along the z-axis compared with the control and mineral-only groups. The reconstructed cross-sectional views revealed that cells infiltrated deeper into the scaffold structure, while cells in the T/α-TCP groups remained predominantly localized near the surface. The false-color depth mapping further confirmed a broader distribution of cells across the z-axis in the fiber-containing group, indicating increased vertical migration and spatial occupancy. These findings demonstrate that the cotton-type nanofibrous architecture provides a three-dimensional permissive environment that facilitates cellular infiltration, thereby complementing its role in guiding cell alignment and enhancing overall cellular responses.

RUNX2 staining further revealed enhanced osteogenic differentiation in the mineral-treated groups compared with the control (Figure 4h). The highest expression was observed in the c-NF@T/α-TCP group. Quantification of MFI further supported this result (Figure 4i), indicating that the integration of bioactive mineral phases with the three-dimensional fibrous architecture effectively promotes early osteogenic commitment.

Overall, these findings demonstrate that while biochemical stimulation from calcium phosphate phases contributes to both angiogenic and osteogenic responses, the presence of a cotton-type nanofibrous architecture provides additional topographical guidance that enhances cell alignment, endothelial activation, and osteogenic differentiation. This synergistic interplay between structural and biochemical cues further supports the effectiveness of the mineral–fiber hybrid strategy in regulating cell behavior.

### 3.5. In Vivo Micro-CT Evaluation of Bone Regeneration in Femoral Defects

**Figure 5:**
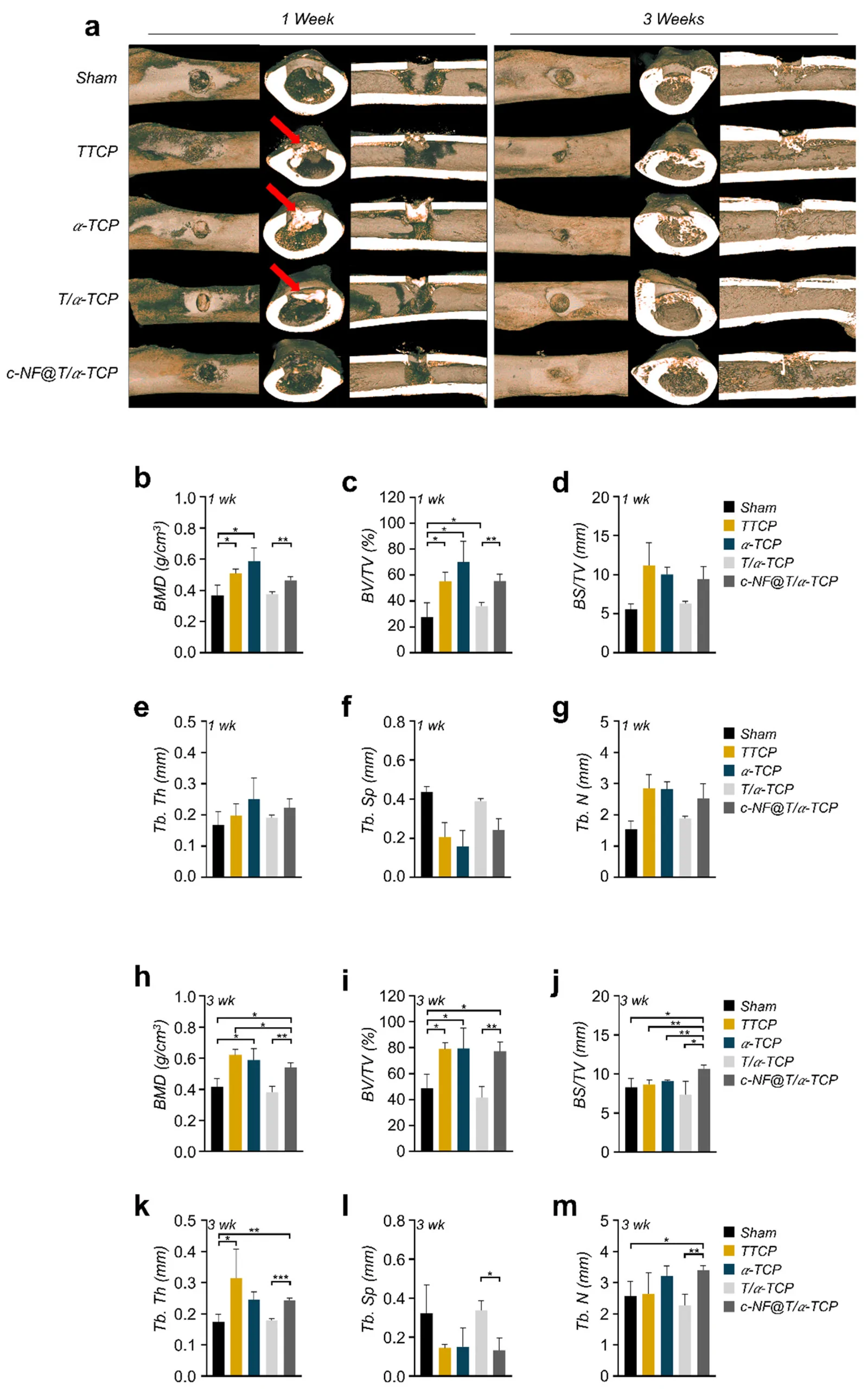
Micro-CT images showing bone healing in femoral defect regions at 1 and 3 weeks post-surgery (n = 3). Red arrows indicate agglomerated mineral particles forming dense clusters within the defect region (**a**). Quantitative micro-CT analysis of bone regeneration, including bone mineral density (BMD), bone volume fraction (BV/TV), bone surface density (BS/TV), trabecular thickness (Tb.Th), trabecular separation (Tb.Sp), and trabecular number (Tb.N) (**b**–**m**). Data are presented as mean ± SD. Statistical significance: * *p* < 0.05, ** *p* < 0.01, *** *p* < 0.001.

Figure 5a presents representative three-dimensional micro-CT reconstructions of rat femoral defect sites at 1 and 3 weeks post-implantation, together with orthogonal cross-sectional views along the longitudinal and transverse planes to visualize spatial bone formation within the defect region. The reconstructed images clearly demonstrate distinct differences in early-stage bone regeneration behaviors depending on the implanted materials. Quantitative micro-CT–based bone morphometric analyses, including bone mineral density (BMD), bone volume fraction (BV/TV), bone surface density (BS/TV), trabecular thickness (Tb.Th), trabecular separation (Tb.Sp), and trabecular number (Tb.N), are summarized in Figure 5b–m. At 1 week post-implantation, dense mineral agglomerates were frequently observed within the defect region in the mineral-only groups. Because these residual particulate clusters exhibited high radiopacity and could be misidentified as newly formed mineralized tissue, the agglomerated mineral regions were manually excluded from the region-of-interest during quantitative analysis. This correction ensured that the morphometric parameters reflected newly regenerated bone rather than retained implant materials. The need for such exclusion further indicates the limited early integration and dispersion of particulate minerals within the defect environment. At both 1 and 3 weeks post-surgery, all mineral-implanted groups (TTCP and α-TCP) exhibited significantly enhanced bone formation compared with the sham control group, confirming the intrinsic osteoconductive nature of calcium phosphate–based materials as shown in Figure 5c,i. In contrast, the biphasic T/α-TCP group demonstrated only marginal improvement, showing bone healing levels that were slightly higher than or comparable to those of the sham group. This relatively limited regenerative response suggests that the biological advantage of combining TTCP and α-TCP is not simply additive and may instead be moderated by altered dissolution kinetics and ionic release behavior in the multiphase system. This trend is consistent with the in vitro findings, where the T/α-TCP condition exhibited reduced cellular activity compared with the single-phase TTCP group (Figure 3b). The mineral-treated defects showed increased BMD and BV/TV, reflecting enhanced mineral deposition and new bone volume within the defect region as presented in Figure 5b,c,h,i. BS/TV and Tb.N were elevated, indicating the formation of a more developed trabecular network, while Tb.Th increased and Tb.Sp decreased over time, suggesting progressive trabecular thickening and densification of the regenerating bone tissue (Figure 5d–g,j–m). Despite the widespread use of biphasic calcium phosphate composites in both preclinical studies and clinical applications, the T/α-TCP composite group did not outperform the single-phase mineral groups in the present defect model. In several morphometric parameters, the composite exhibited comparable or slightly inferior regenerative outcomes relative to TTCP or α-TCP alone. This observation suggests that, in the absence of a structural framework facilitating spatial dispersion and cellular infiltration, the mere compositional combination of two calcium phosphate phases does not necessarily guarantee synergistic enhancement of early bone regeneration. The performance of mineral-only implants appears to be constrained by their limited ability to spatially distribute within the defect site and to provide continuous guidance cues for host tissue invasion.

The spatial distribution of implanted materials within the defect site provides mechanistic insights into these observations. As shown in the 1-week micro-CT images (Figure 5a), mineral-only powders remained largely agglomerated within the defect region, forming dense clusters that were spatially confined and partially embedded within surrounding soft tissues. This localized aggregation suggests that the mineral particles were mechanically compacted during implantation and remained poorly dispersed during the early post-implantation period. Gross inspection at the time of 1-week specimen harvesting further revealed that the implanted mineral powders largely retained their particulate form within the defect cavity, indicating delayed integration with host tissues. Such agglomeration is expected to hinder early tissue penetration and vascular invasion, thereby imposing kinetic limitations on cell recruitment and subsequent bone formation. Consequently, mineral-only constructs may promote bone regeneration in a delayed and spatially restricted manner, governed primarily by slow tissue ingrowth from the defect periphery. In contrast, the c-NF@T/α-TCP scaffold exhibited markedly different early-stage regenerative behavior. No obvious mineral agglomerates were observed within the defect site as early as 1 week post-implantation, indicating that the T/α-TCP particles were spatially dispersed along the cottony nanofibrous framework rather than forming dense clusters. This homogeneous spatial distribution of mineral cues throughout the defect volume is attributed to the fibrous architecture of the c-NF scaffold, which acts as a physical pathway enabling deep penetration of host tissues and rapid cellular infiltration. The interconnected macroporous network of the cottony nanofibers provides continuous conduits for cell migration and neovascular ingrowth, thereby facilitating early access of osteogenic cells to mineralized surfaces distributed throughout the defect site as shown in Figure 5b–m [58,59,60]. Consistent with these structural advantages, the c-NF@T/α-TCP group exhibited the most pronounced enhancement across all evaluated bone morphometric parameters, including BMD, BV/TV, BS/TV, Tb.Th, Tb.Sp, and Tb.N, at both 1 and 3 weeks post-implantation. The simultaneous increase in trabecular number and thickness, together with the reduction in trabecular separation, indicates accelerated formation of a dense and well-connected trabecular network within the defect region. These results suggest that the integration of biphasic calcium phosphate minerals into a three-dimensional cottony nanofibrous scaffold effectively overcomes the spatial and kinetic limitations inherent to mineral-only implants by providing fibrous pathway–mediated access for host cells and vasculature.

These micro-CT findings demonstrate that while calcium phosphate minerals alone possess intrinsic osteoconductive potential, their regenerative efficacy in vivo is strongly influenced by their spatial presentation and accessibility within the defect environment. The incorporation of T/α-TCP into a highly porous, interconnected fibrous network enables homogeneous mineral distribution and early tissue infiltration, thereby promoting more rapid and robust bone regeneration compared with mineral-only constructs. This highlights the critical role of scaffold architecture, beyond material composition alone, in dictating the kinetics and quality of in vivo bone healing.

### 3.6. Histological and Histomorphometric Evaluation of Bone Regeneration and Neovascularization

**Figure 6:**
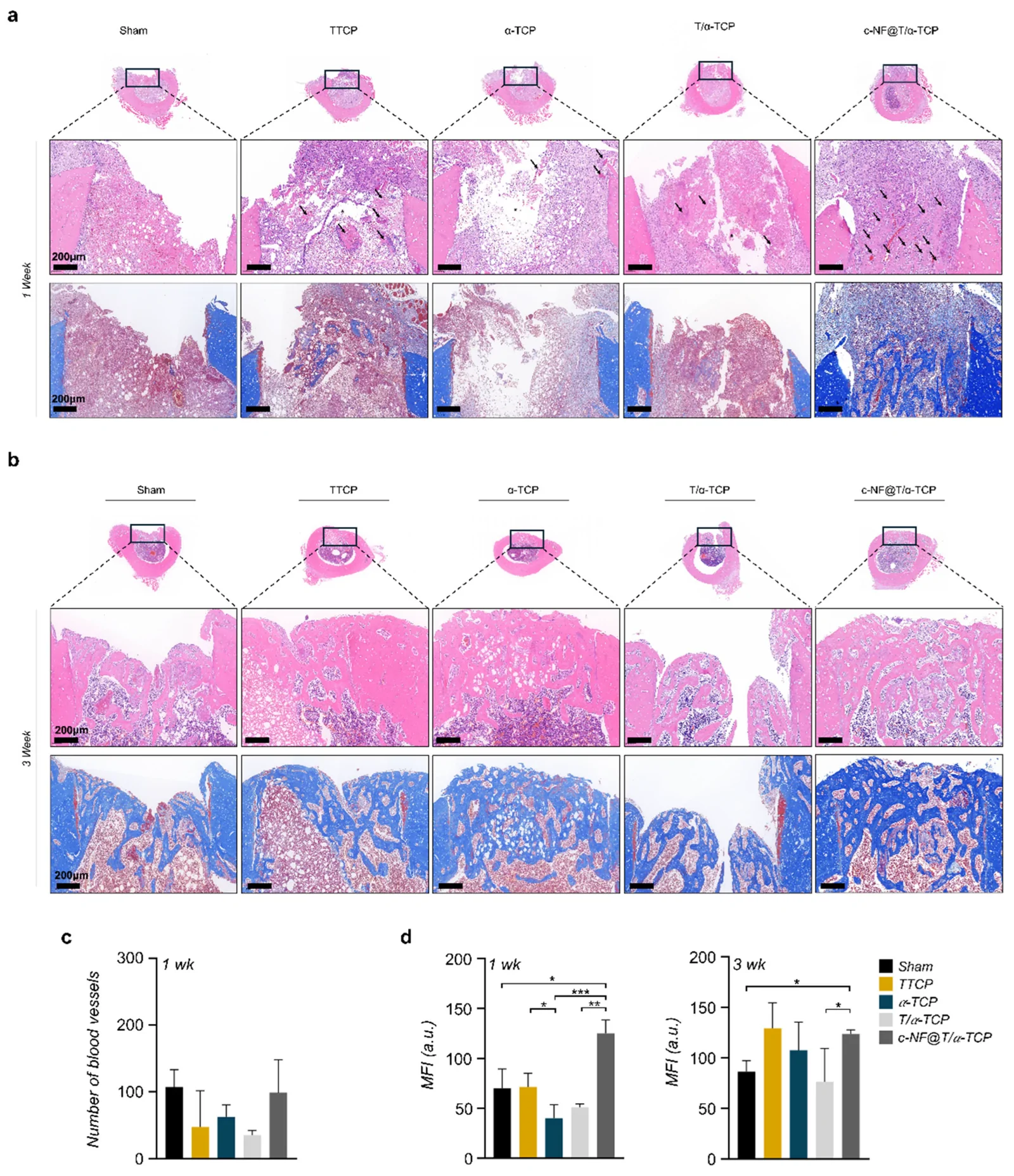
Representative H&E and Masson’s trichrome (MTS) stained images of femoral defect regions at 1 week (**a**) and 3 weeks (**b**) post-surgery (n = 3), where black arrows in (**a**) indicate areas of newly formed bone within the defect region. Quantification of H&E (**c**) and MTS (**d**) staining. Data are presented as mean ± SD. Statistical significance: * *p* < 0.05, ** *p* < 0.01, *** *p* < 0.001.

Figure 6a presents representative hematoxylin and eosin (H&E) and Masson’s trichrome staining (MTS) images of femoral defect sites harvested at 1 and 3 weeks post-implantation. Consistent with the micro-CT observations as shown in Figure 5, distinct differences in early-stage tissue responses were evident depending on the scaffold configuration. At 1 week post-surgery, prominent mineral pockets (asterisks denote mineral pockets in the 1-week H&E sections) were observed in the TTCP-, α-TCP-, and T/α-TCP–treated defects (Figure 6a). These mineral aggregates remained locally confined within the defect region and were surrounded by fibrous or soft connective tissues, with limited evidence of direct tissue penetration into the mineral clusters (Figure 6a). In contrast, newly formed bone tissues (arrows indicate newly formed bone in the 1-week H&E images) were sparsely distributed in these mineral-only groups at this early time point, suggesting delayed osteointegration and limited early-stage tissue infiltration. However, defects treated with c-NF@T/α-TCP exhibited abundant newly formed bone tissue as early as 1 week post-implantation. H&E staining revealed extensive infiltration of host tissues into the defect region, accompanied by early trabecular bone formation adjacent to and along the fibrous scaffold as shown in Figure 6a. This early osteogenic response was more clearly visualized in the MTS images, which demonstrated pronounced collagen deposition and matrix organization within the defect space (Figure 6b). The blue-stained collagen-rich regions in the MTS sections indicate active extracellular matrix remodeling and early-stage bone matrix formation in the c-NF@T/α-TCP group, highlighting the capacity of the fibrous pathway–enabled scaffold to accelerate early tissue ingrowth and osteoid deposition. At 3 weeks post-implantation, all mineral-treated groups, including TTCP, α-TCP, T/α-TCP, and c-NF@T/α-TCP, exhibited substantial new bone formation within the defect regions, with markedly higher bone regeneration compared with the sham group as shown in Figure 6d. Newly formed trabecular bone structures were observed bridging the defect area, accompanied by increased collagen matrix deposition in the MTS-stained sections. These results confirm the intrinsic osteoconductive nature of calcium phosphate–based materials. However, consistent with the early-stage findings, the c-NF@T/α-TCP group exhibited the most extensive bone formation and the most advanced collagen remodeling, indicating a sustained advantage in both the rate and quality of bone regeneration relative to mineral-only constructs. To further elucidate the mechanistic basis underlying the enhanced regenerative performance of c-NF@T/α-TCP, neovascularization within the defect region was quantitatively assessed based on H&E-stained sections as presented in Figure 6c. Blood vessels in the peri-defect region were identified morphologically and counted to evaluate early angiogenic responses. Quantitative analysis revealed a significantly higher density of blood vessels in the c-NF@T/α-TCP–treated defects compared with the TTCP, α-TCP, and T/α-TCP groups at 1 week post-implantation. Vascular structures were rarely observed within or adjacent to the mineral pocket regions in the mineral-only groups, indicating that densely aggregated mineral clusters act as physical barriers that impede vascular invasion and tissue penetration. The absence of vascular structures within these mineral pockets suggests that early angiogenesis is spatially restricted, which likely contributes to delayed cellular recruitment and slower initiation of bone regeneration in these groups. In contrast, the c-NF@T/α-TCP scaffold facilitated uniform spatial distribution of mineral cues along the three-dimensional fibrous network, thereby eliminating the formation of large mineral agglomerates and enabling continuous pathways for tissue and vascular ingrowth. The interconnected macroporous architecture of the cottony nanofibers provides permissive conduits for endothelial cell migration and vessel sprouting, promoting rapid neovascularization throughout the defect volume. Given the well-established coupling between angiogenesis and osteogenesis during bone repair, the enhanced early vascularization observed in the c-NF@T/α-TCP group provides a mechanistic explanation for the accelerated bone formation and collagen remodeling seen in both histological and micro-CT analyses. These histological and histomorphometric results corroborate the micro-CT findings and demonstrate that mineral-only powders, although osteoconductive, tend to form localized mineral pockets that hinder early tissue and vascular infiltration, thereby delaying the initiation of robust bone regeneration. In contrast, the fibrous pathway–enabled c-NF@T/α-TCP scaffold overcomes these spatial limitations by providing a highly porous, interconnected framework that supports rapid host tissue invasion and neovascularization, ultimately leading to enhanced early-stage bone formation and more mature collagen matrix remodeling.

### 3.7. Immunohistochemical Evaluation of Angiogenesis and Osteogenic Differentiation

**Figure 7:**
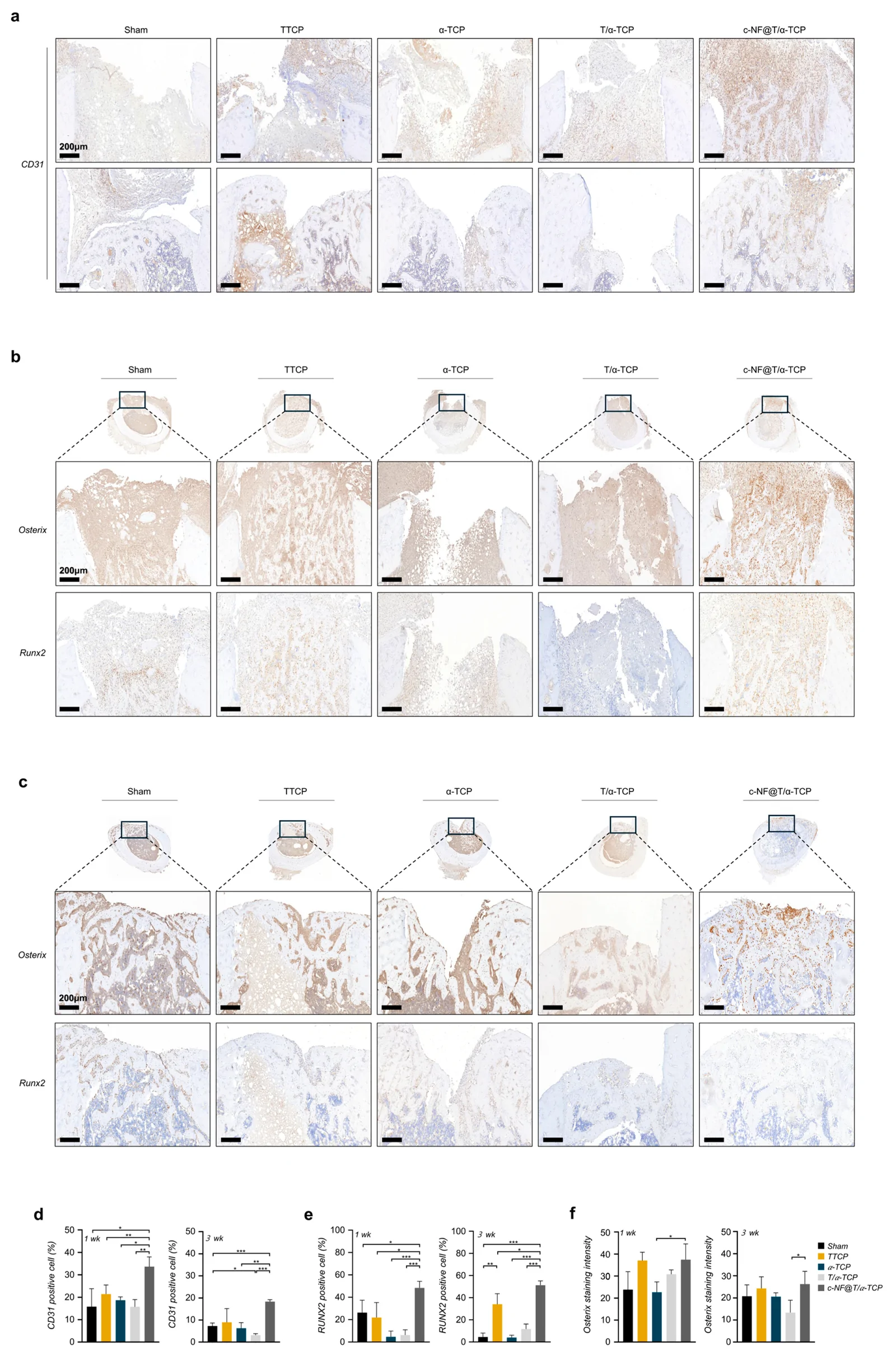
Immunohistochemical (IHC) images of CD31 expression in femoral defect regions at 1 week and 3 weeks post-surgery (**a**). IHC images showing Osterix and RUNX2 expression in femoral defect regions at 1 week (**b**) and 3 weeks (**c**) post-surgery. Quantification of CD31-positive cells (**d**) and RUNX2-positive cells (**e**) and Osterix staining intensity (**f**) in each group. Data are presented as mean ± SD. Statistical significance: * *p* < 0.05, ** *p* < 0.01, *** *p* < 0.001.

To further evaluate vascularization within the defect region, immunohistochemical (IHC) staining for CD31 was performed at 1 and 3 weeks post-implantation (Figure 7a). CD31 is a representative endothelial marker indicative of microvessel formation and angiogenic activity. At 1 week, the mineral-only groups exhibited relatively limited CD31-positive staining, suggesting restricted vascular infiltration within the defect area. In particular, the T/α-TCP group showed sparse and discontinuous vessel-like structures, In particular, the T/α-TCP group showed sparse and discontinuous vessel-like structures, likely due to the formation of dense mineral aggregates that limit early tissue and vessel infiltration. In contrast, the c-NF@T/α-TCP group displayed markedly enhanced CD31-positive staining, with well-distributed vessel-like structures observed throughout the defect region. This pronounced increase indicates that the cotton-type nanofibrous architecture facilitates rapid vascular ingrowth during the early healing phase. At 3 weeks, CD31 expression increased across all groups; however, the c-NF@T/α-TCP group maintained the highest level of vascularization, showing more extensive and continuous microvessel formation compared to the other conditions. Quantitative analysis of CD31-positive cells (%) further confirmed these observations, demonstrating a significantly higher blood vessel density in the c-NF@T/α-TCP group at both time points (Figure 7d). These findings indicate that the cotton-type nanofiber–integrated scaffold effectively enhances vascular infiltration within the defect site. The enhanced angiogenic response is likely governed by a synergistic mechanism involving both physical and biochemical cues. The interconnected fibrous network provides permissive pathways for vessel invasion, while the calcium phosphate phases contribute biochemical stimulation through ionic release. Together, these effects promote rapid and sustained vascularization, supporting subsequent tissue regeneration.

In addition, To further investigate the early osteogenic commitment within the defect region, IHC staining for RUNX2 and osterix (OSX) was performed at 1 and 3 weeks post-implantation (Figure 7b,c). These transcription factors represent key regulators of osteoblast lineage progression, where RUNX2 is associated with early osteoprogenitor activation and OSX reflects subsequent osteoblast maturation. At both time points, the TTCP-treated defects exhibited markedly elevated RUNX2 expression compared with the other mineral-only groups (Figure 7e). The strong RUNX2-positive staining observed at 1 week indicates rapid recruitment and early commitment of osteoprogenitor cells, which remained evident at 3 weeks, suggesting sustained osteogenic activity. This enhanced transcriptional activation is consistent with the in vitro findings showing higher ALP activity in the TTCP group, as well as with the increased new bone formation and mineral deposition observed in the micro-CT and histological analyses (Figure 3b, Figure 5 and Figure 6). These results confirm that TTCP provides a strong biochemical stimulus capable of accelerating early osteogenic differentiation in vivo. However, the biphasic T/α-TCP group showed noticeably lower RUNX2 expression at both 1 and 3 weeks (Figure 7e). This attenuated response suggests that the osteogenic stimulus of TTCP may be partially moderated within the multiphase system, likely due to altered dissolution kinetics and reduced ionic supersaturation. The reduced RUNX2 activation in this group is in good agreement with its relatively limited cellular activity in vitro and the marginal early bone formation observed in the micro-CT analysis, indicating that compositional complexity alone does not necessarily enhance early osteogenic signaling. The c-NF scaffold alone exhibited minimal RUNX2 expression, reflecting the limited osteoinductive capacity of the polymeric fibrous structure in the absence of mineral-derived biochemical cues. However, when the biphasic minerals were integrated within the cotton-type fibrous architecture (c-NF@T/α-TCP), a pronounced increase in RUNX2-positive cells was observed at both time points (Figure 7e). The level of RUNX2 expression in this group was substantially higher than that of the T/α-TCP mineral-only condition. This enhancement is likely associated with the improved early tissue infiltration and cell–material interaction enabled by the interconnected cottony network, which facilitates rapid access of host progenitor cells to the mineralized surfaces.

A similar trend was observed in the expression of osterix (OSX), which reflects progression toward osteoblastic maturation as shown in Figure 6a,b. TTCP demonstrated the highest OSX expression at both 1 and 3 weeks, whereas α-TCP showed a moderate level and the T/α-TCP composite exhibited the lowest expression among the mineral-only groups (Figure 7f). The c-NF scaffold alone displayed weak OSX staining, consistent with its limited osteogenic stimulation. In contrast, the c-NF@T/α-TCP group showed a marked increase in OSX-positive cells compared with the biphasic mineral-only condition, indicating enhanced osteoblast differentiation and matrix-forming activity within the defect region. The concordant upregulation of both RUNX2 and OSX in the c-NF@T/α-TCP group highlights the critical role of early structural integration in regulating osteogenic signaling. By preventing mineral aggregation and providing continuous pathways for host cell infiltration, the cotton-type nanofiber architecture enables more effective spatial presentation of osteoconductive cues, thereby amplifying the biological response of the incorporated minerals. These findings suggest that early tissue ingrowth, facilitated by the fibrous pathway structure, is a key determinant of transcriptional activation and subsequent bone regeneration.

The IHC results are well aligned with the in vitro assays, micro-CT morphometric data, and histological observations, demonstrating that while TTCP provides strong intrinsic osteogenic stimulation, the integration of biphasic minerals within a cotton-type fibrous framework restores and enhances early osteogenic signaling by improving tissue accessibility and cellular recruitment. This further supports the concept that scaffold architecture, in addition to material composition, plays a decisive role in governing early bone regeneration dynamics.

## 4. Conclusions

In this study, we demonstrate that the regenerative efficacy of mineral-based bone grafts is governed not only by their intrinsic osteoconductivity, but critically by their early-stage structural presentation, which determines the accessibility of mineral phases to host tissues. By introducing a cotton-type nanofiber–guided pathway architecture, we achieved homogeneous mineral dispersion and continuous tissue-ingrowth pathways, thereby overcoming the early integration limitations of conventional particulate grafts. This structural strategy promotes rapid cellular infiltration and vascularization, ultimately enhancing bone regeneration by improving early microenvironmental accessibility. Our findings highlight that scaffold architecture, rather than compositional complexity alone, plays a decisive role in early-stage regenerative outcomes. In addition, this work establishes fibrous pathway engineering as a simple and scalable design principle for reprogramming particulate biomaterials, offering a versatile platform to bridge the gap between osteoconductivity and reliable early-stage tissue integration.

## 5. Statistical Analysis

Data are presented as mean ± standard error of the mean (SEM). For comparisons between two groups, statistical significance was evaluated using an unpaired two-tailed Student’s *t*-test. For multiple group comparisons (≥3 groups), one-way ANOVA followed by Tukey’s post hoc test was applied. A *p*-value < 0.05 was considered statistically significant. Significance levels were denoted as * *p* < 0.05, ** *p* < 0.01, and *** *p* < 0.001 versus the control group, unless otherwise specified.

## Data Availability

The original contributions presented in the study are included in the article, further inquiries can be directed to the corresponding authors.
